# Channeling Vision: Ca_V_1.4—A Critical Link in Retinal Signal Transmission

**DOI:** 10.1155/2018/7272630

**Published:** 2018-05-09

**Authors:** D. M. Waldner, N. T. Bech-Hansen, W. K. Stell

**Affiliations:** ^1^Department of Neuroscience, Cumming School of Medicine, University of Calgary, Calgary, AB, Canada; ^2^Department of Medical Genetics and Department of Surgery, Alberta Children's Hospital Research Institute, and Hotchkiss Brain Institute, Cumming School of Medicine, University of Calgary, Calgary, AB, Canada; ^3^Department of Cell Biology and Anatomy and Department of Surgery, Hotchkiss Brain Institute, and Alberta Children's Hospital Research Institute, Cumming School of Medicine, University of Calgary, Calgary, AB, Canada

## Abstract

Voltage-gated calcium channels (VGCC) are key to many biological functions. Entry of Ca^2+^ into cells is essential for initiating or modulating important processes such as secretion, cell motility, and gene transcription. In the retina and other neural tissues, one of the major roles of Ca^2+^-entry is to stimulate or regulate exocytosis of synaptic vesicles, without which synaptic transmission is impaired. This review will address the special properties of one L-type VGCC, Ca_V_1.4, with particular emphasis on its role in transmission of visual signals from rod and cone photoreceptors (hereafter called “photoreceptors,” to the exclusion of intrinsically photoreceptive retinal ganglion cells) to the second-order retinal neurons, and the pathological effects of mutations in the* CACNA1F* gene which codes for the pore-forming α_1F_ subunit of Ca_V_1.4.

## 1. History

The original discovery of Ca_V_1.4 is intimately associated with the phenotypic and genotypic characterization of a family of inherited retinal disorders, congenital stationary night blindness (CSNB), a subset of which (CSNB2A) is caused by mutations in* CACNA1F. *CSNB first appeared in historical records in the 17th century associated with a Frenchman named Jean Nougaret for whom an extensive genealogical pedigree was available, implicating the disease as an autosomal dominant inherited condition [1]. Additional cases of hereditary night blindness with recessive and X-linked inheritance patterns were reported subsequently [2–5]. Widespread clinical use and understanding of electroretinography (ERG) in the mid-20th century allowed clinicians to begin separating CSNB into distinct clinical entities. Schubert and Bornschein first reported a subtype of CSNB in which the scotopic, negative-going a-wave was normal, while the positive-going b-wave was significantly attenuated [6]. CSNB with this electronegative ERG response (i.e., with a b-wave significantly smaller in amplitude than the a-wave) would be known thereafter as “Schubert-Bornschein-type CSNB,” to distinguish it from “Riggs-type CSNB,” in which both a- and b-waves exhibit decreased amplitude [7]. Phenotypic heterogeneity of individuals with Schubert-Bornschein-type CSNB led Miyake to separate the disorder into “complete” (with* total* loss of rod function) and “incomplete” (with* partial* rod function) subtypes in 1986 [8]. This insight would subsequently be validated genetically, following comprehensive linkage analysis, with distinct gene loci identified for the causative mutations in “complete” and “incomplete” X-linked CSNB [9]. Large-scale DNA sequencing of Xp11.23, the genomic region implicated in X-linked incomplete CSNB (CSNB2A), led to the simultaneous identification by two research groups of mutations in the recently discovered gene “*CACNA1F” *in individuals with the condition [10, 11].* CACNA1F *had been annotated in a publication only one-year prior, where segments of sequence were recognized as having high homology to known VGCC pore-forming *α*_1_ subunits [12].* CACNA1F* is now known to code for *α*_1F_, the primary subunit of the voltage-gated L-type calcium channel Ca_V_1.4.

## 2. Molecular Properties, Distribution, and Function

### 2.1. *CACNA1F*, *α*_1F_ , and  Ca_V_1.4

**Figure 1:**
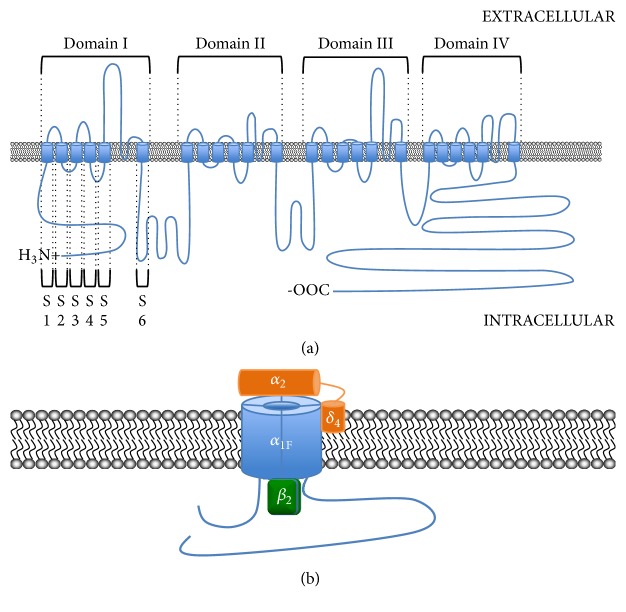
(a) Protein topology of Ca_V_1.4  *α*_1F_ subunit. (b) Schematic of Ca_V_1.4, including accessory subunits *β*_2_ and *α*_2_*δ*_4_.

*CACNA1F* is a 48 exon gene encoding *α*_1F_, a 1977 amino-acid protein with the distinct structural characteristics typical of VGCC *α*_1_ subunits [11, 13, 14]. These include intracellular N- and C-termini flanking four homologous domains, each of which consists of six transmembrane alpha helix segments (Figure 1(a); for a review of voltage-gated calcium channels, see Zamponi et al., 2015 [15]). *α*_1F_ is one of ten human *α*_1_ VGCC subunits, which are the primary determinants of Ca_V_ channel characteristics [16]. In addition to the pore-forming *α*_1_ subunits, VGCCs are also composed of several other accessory subunits that may differ in subtype, altering channel-trafficking, kinetics, and other properties. These include *β*, *α*_2_*δ*, and *Υ* subunits, which, together with *α*_1_, comprise VGCCs in a 1 : 1 : 1 : 1 ratio [16] (Figure 1(b)). By conventional nomenclature VGCCs are named for their *α*_1_ subunit; all VGCCs that have *α*_1F_ as their pore-forming subunit are classified as Ca_V_1.4 channels, though the accessory subunits may differ [16].* In vivo, *Ca_V_1.4 channels have been shown to primarily include the subunit subtypes *β*_2_ and *α*_2_*δ*_4_ [17–21]. Gamma (*Υ*) subunits in Ca_V_1.4, if present, have not yet been identified.

### 2.2. Ca_V_1.4 Distribution and Expression

Ca_V_1.4 is primarily expressed in the retina, though unique splice variants of* CACNA1F (*with significantly different functions) are also expressed in B- and T-lymphocytes and possibly other immune cells [10, 22]. Broader tissue expression of Ca_V_1.4 has been suggested from analysis of expressed sequence tags (EST), but immunohistochemical experiments have largely failed to confirm these suggestions [23]. The apparent presence of the channel in these unconfirmed locations might be due to the presence of circulating immune cells in these tissues when they were sampled for EST analysis [24]. Furthermore, even in the retina some localizations of Ca_V_1.4 remain controversial. Early reports identified *α*_1F_–like immunoreactivity and* Cacna1f* mRNA in the inner, as well as the outer, plexiform layer [13, 25, 26], whereas more recent immunohistochemical evidence suggests exclusive expression in photoreceptor terminals of the OPL [27–30]. Recent electrophysiological recordings of bipolar cell axon terminals of the IPL in* Cacna1f*-deficient mouse lines suggest that Ca_V_1.4 contributes slightly to calcium influx in those cells, though this may be due to alterations in retinal circuitry or bipolar cell homeostasis resulting from dysfunctional photoreceptor synaptic transmission [31]. Thus, the localization of Ca_V_1.4 beyond the photoreceptors of the retina remains to be resolved definitively, due in part to difficulties in creating subtype-specific antibodies or possibly very low-level protein expression in bipolar cell terminals.

### 2.3. Ca_V_1.4 Function: The Ribbon Synapse and Phototransduction

**Figure 2:**
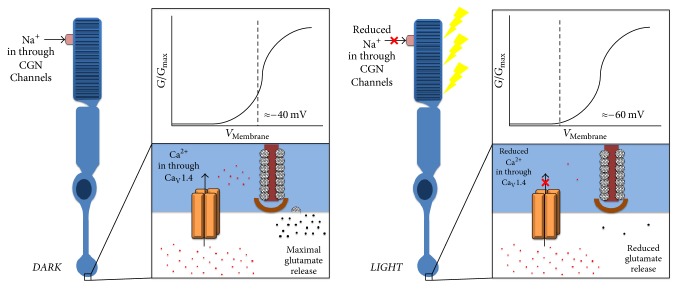
Effect of phototransduction on photoreceptor membrane potential, calcium influx through Ca_V_1.4 and glutamate release at the ribbon synapse. In complete darkness, sodium ion influx through open CGN channels in photoreceptor outer segments maintains the membrane potential at ~−40 mV, where channel conductance (*G*/*G*_max_) and glutamate release are at their physiological maxima. Phototransduction in response to increased illumination causes graded decreases in sodium ion influx through CGN channels, a hyperpolarization of the photoreceptor membrane, a decrease in calcium ion influx into the presynaptic terminal and resultant decreased glutamate release.

The retina-specific expression of Ca_V_1.4 and the visual dysfunction resulting from its disruption (CSNB2A) allude to the crucial role of this particular channel in visual signal transduction. At photoreceptor terminals, Ca_V_1.4 channels are an integral component of the characteristic “ribbon synapses,” which are responsible for neurotransmission at photoreceptor and bipolar cell synapses. Synaptic ribbons are electron-dense, specialized protein complexes that recruit large numbers of glutamate-filled vesicles near the synaptic terminal, allowing for sustained vesicular release in response to sustained calcium influx [32, 33]. Unlike conventional neurons, rod and cone photoreceptors exhibit tonic glutamate release with maximal exocytosis in complete darkness and a graded decrease with increasing illumination. The phototransduction cascade is such that absorption of a photon within a photoreceptor outer segment causes a relative hyperpolarization (decrease in sustained depolarization) of the photoreceptor cell membrane, thereby decreasing the activation of the voltage-sensitive Ca_V_1.4 channels and lessening calcium influx within the presynaptic terminal (Figure 2; for a review of phototransduction, see Yau, 1994 [34]). The proper assembly and function of the ribbon synapse involves a multitude of proteins and protein-protein interactions, which, if disrupted, can cause a loss of visual function (for a review of protein constituents and interactions of the photoreceptor ribbon synapse, see Mercer and Thoreson, 2011 [35]). The unique coupling of phototransduction and the ribbon synapse is responsible for the extraordinary sensitivity of the visual system and requires the unique properties of Ca_V_1.4 for optimal function.

### 2.4. Electrophysiology of Photoreceptors and Heterologous Ca_V_1.4 Expression Systems

Before Ca_V_1.4 had been discovered and established as the functional calcium channel of photoreceptor terminals, electrophysiological recordings in nonmammalian photoreceptor inner segments (used for their comparatively large size) had revealed calcium currents with distinctive dynamics (reviewed in Doering et al., 2007 [24]). *I*_ca_ was shown to activate rapidly above roughly −45 to −40 mV, show sensitivity to typical L-type channel agonists and antagonists, and exhibit comparatively little voltage-dependent inactivation and no calcium-dependent inactivation (CDI) under typical recording conditions (although slight CDI was observable with reduced EGTA calcium buffering) [41, 42]. Many neuromodulators were shown to influence this photoreceptor calcium current, via mechanisms that remain largely unresolved today (for a review of these modulatory compounds and their effects, see Krizaj and Copenhagen 2002 [43]).

Following the identification of the* CACNA1F* gene, electrophysiological recordings in heterologous systems expressing Ca_V_1.4 verified it as the channel mainly responsible for the unique calcium current observed in these earlier studies. The biophysical properties of Ca_V_1.4 in these expression systems have been extensively reviewed [24] and so will be mentioned only briefly here. *α*_1F_ transiently expressed in human embryonic kidney cells with accessory subunits *β*_3/2a_ and *α*_2_*δ*_1_ form a Ca_V_1.4 channel that (1) is activated near −40 mV, the resting membrane potential of photoreceptors [23, 44, 45]; (2) exhibits the smallest unitary conductance of all known VGCCs, and an exceptionally low open probability [46]; (3) is inactivated as a function of voltage (voltage-dependent inactivation, recorded with Ba^2+^ as charge-carrier) more slowly than Ca_V_1.2 and Ca_V_1.3 [23, 44, 45]; and (4) exhibits almost no calcium-dependent inactivation [23, 44, 45, 47]. These distinctive properties are all essential to maintain the constant calcium influx without inactivation that is necessary for sustained glutamate release from rods and cones.

## 3. The Complexity of the Photoreceptor Calcium Current and Ca_V_1.4 In Vivo

While heterologous systems are valuable for determining basic kinetic properties of Ca_V_1.4, none of them has yet captured fully the complexities of the photoreceptor calcium current.* In vivo, *retinal photoreceptors express simultaneously several splice isoforms of Ca_V_1.4 with different kinetics [48, 49] and associate with unique modulatory subunits (discussed below: Ca_V_1.4* Subunit Composition*) [17]. Additional factors, including physiological temperature (37°C) [50], calmodulin (CaM) coexpression [51–53], and calcium-binding protein 4 (CaBP4) coexpression [54, 55], also significantly modulate biophysical parameters of the channel. Thus, descriptions of Ca_V_1.4 in all heterologous expression systems to date likely differ significantly from those of the channel as it exists* in vivo*.

### 3.1. *CACNA1F* Splice Isoforms

Over 20 splice isoforms of* CACNA1F* have been identified, several of which have significantly disparate dynamics [48, 49]. Most remarkably, several splice variants exist in which the C-terminus is truncated or otherwise disrupted. Tan et al. identified a splice variant incorporating an alternate 43rd exon, containing a stop codon that truncates ~1/2 of the C-terminus and represents 13.6% of the total* CACNA1F* transcript identified in their screening experiment of a human retina cDNA library [49]. Haeseleer et al. additionally identified two transcripts in which the C-terminal 47th exon is absent [48]. Investigations of the biophysical properties of the Ca_V_1.4 isoforms encoded by these transcripts revealed significant differences from the wild-type, including a hyperpolarizing shift in activation voltage (compared to the full-length channel), and robust calcium-dependent inactivation. These data support models of ICDI/CTM regulation of Ca_V_1.4 (discussed below:* C-Terminal Regulation and Calmodulin*) and suggest that coexpression of alternative splice variants results in a complex calcium current in photoreceptor terminals* in vivo*.

In extraretinal locations, unique splice variants of* CACNA1F* have also been identified in T-lymphocytes, which substantially alter the topology and function of the Ca_V_1.4 channel. One of these in particular lacks a significant portion of the C-terminus and domain IV (including this domain's voltage-sensing segment) making this isoform unlikely to function as a voltage-gated channel. These channels have therefore been proposed to function via alternative gating mechanisms, likely via direct intracellular signaling from T-cell receptors [22]. Investigations in* Cacna1f *knockout mice have shown that Ca_V_1.4 is critical for the survival, proliferation, and signaling of CD4^+^ and CD8^+^ T-cells, suggesting an essential role for these unique splice isoforms in normal functioning of the immune system [56].

### 3.2. Ca_V_1.4 Subunit Composition

As previously mentioned, *α*_1F_ associates* in vivo* with *β*_2_ and *α*_2_*δ*_4_ subunits, which regulate and modulate the calcium conductance of Ca_V_1.4 [17–21]. The predominant *β*_2_ splice variant in retinal Ca_V_1.4 complexes, *β*_2X13_, is unique in that it includes the N-terminal palmitoylation sites found in *β*_2a_, but an alternate exon 7 (7B instead of 7A, found in *β*_2a_) results in a shorter HOOK domain, associated with modulating VGCC inactivation kinetics [57]. This variant has been shown to increase voltage-dependent inactivation (VDI) of the Ca_V_1.4 channel, in contrast with channels including the *β*_2a_ subunit, although the physiological significance of this effect is unclear [17]. Ca_V_1.4 channels that include the *α*_2_*δ*_4_ subunit have been shown to exhibit more positive voltage-dependent activation and a lack of calcium-dependent facilitation of currents, compared to channels including the *α*_2_*δ*_1_ subunit [17]. Subunit complex *α*_2_*δ*_4_ has recently been shown to regulate the trafficking of *α*_1F_ subunits to the presynaptic membrane, thus regulating channel and therefore calcium current density in the synaptic terminal. Further, *α*_2_*δ*_4_ was shown to be critical for the formation of synapses between rod (but not cone) photoreceptors and the signal relaying ON-bipolar cells of the inner retina (see* Physiological and Morphological Observations in CSNB2A Model Retinas*) [58]. These data suggest, as expected, that the auxiliary subunit composition of Ca_V_1.4 is important in shaping its unique biophysical properties.

### 3.3. C-Terminal Regulation and Calmodulin

The absence of calcium-dependent inactivation (CDI) observed in early characterizations of Ca_V_1.4 suggested that the channel differed from other L-type VGCCs in C-terminal regulation [23, 44, 45]. CDI in Ca_V_1.2 channels has been shown to be a function of the proximal C-terminal EF-hand, Pre-IQ and IQ motifs, which are necessary for interaction with the calcium-binding protein, calmodulin (CaM). In this model, the Pre-IQ and/or IQ domains are constitutively associated with apoCaM, which undergoes a conformational change upon binding calcium ions when they enter the channel pore. This conformational rearrangement of CaM is relayed to the channel core via the EF-hand motif and then inactivates the VGCC, possibly through interaction with the I-II intracellular linker [59–63]. The lack of CDI in Ca_V_1.4 channel was initially confounding, as Ca_V_1.4 exhibits high sequence homology with other Ca_V_1 channels in these proximal C-terminal motifs [64–66].

In 2006, two groups independently showed that the relative dearth of CDI in Ca_V_1.4, in heterologous expression systems, is a function of the distal C-terminus which functions as an autoinhibitory domain. The distal C-terminus was shown via fluorescence resonance energy transfer (FRET) experiments to interact directly with regions of the proximal C-terminus that are necessary for their CDI-mediating interaction with CaM. Singh et al. showed that this interaction also caused a positive shift in the voltage-dependence of activation and thus named the distal C-terminal region the “C-terminal modulator” (CTM), in contrast to the “inhibitor of calcium-dependent inactivation” (ICDI) term introduced by the concurrent discoverers, Wahl-Schott et al. [47, 65].

Whether the ICDI/CTM prevented CDI by competing with CaM binding directly, or by allosterically inhibiting transduction of CaM-mediated conformational changes in the inactivation machinery was not initially clear. Subsequent research has shown that the ICDI/CTM does indeed compete with CaM for occupancy within the proximal C-terminal domains, but incompletely, such that physiological changes in CaM levels within the cell are functionally relevant [52]. The preassociation of apoCaM with the proximal C-terminal domains of Ca_V_1.3 and chimeric Ca_V_1.3/1.4 channels modulates channel properties, causing an increased channel open probability and slowed voltage-dependent inactivation [51, 53]; therefore, variability of expression of CaM in competition with the ICDI/CTM domain likely shapes the population dynamics of the Ca_V_1.4 calcium current within a cell, both by apoCaM modulation and CaM-mediated CDI in bound channels [52].

Additional complexity is added by the recently discovered protein kinase A phosphorylation of the Ca_V_1.4 ICDI/CTM domain. Phosphorylation of S1883 prevents ICDI/CTM interaction with the IQ domain, allowing CaM to bind and thereby increase channel open probability and confer CDI [67]. As PKA activity is dopamine-dependent, and retinal dopamine release is increased by illumination, Ca_V_1.4 phosphorylation may be an important physiological mechanism for the regulation of visual sensitivity [68–71]. As this scheme has only recently been identified in a heterologous expression system, additional research is expected to provide further insight into functional consequences* in vivo*.

### 3.4. Calcium-Binding Protein 4 (CaBP4)

The function of a second protein modulator, calcium-binding protein 4 (CaBP4), also appears to be essential for Ca_V_1.4 function* in vivo. *CaBP4 is a one member of a subfamily of calmodulin-like proteins implicated in the regulation of voltage-gated calcium channels and inositol triphosphate receptors [72–74]. Mutations in CaBP4 cause autosomal recessive CSNB2 in humans, and mouse CaBP4-KO models exhibit a retinal phenotype similar to that of Ca_V_1.4-KO mice [55, 75]. Research has shown that CaBP4 also binds to the C-terminus of the Ca_V_1.4 channel, and in ICDI/CTM-truncation mutants it prevents CaM-mediated CDI [54, 55]. When ICDI/CTM is present, CaBP4 hyperpolarizes the channel-activation voltage, countering the positive shift attributed to the ICDI/CTM itself, and thus allowing Ca_V_1.4 to function optimally at the physiological photoreceptor membrane potential [54]. Despite this apparent neutralization, both CaBP4 and the ICDI/CTM domain are necessary for proper channel function, as deleterious mutations in either one of them independently causes CSNB2 (see below:* CSNB2-Causing Mutations*). This suggests that the distal C-terminus may have an additional, essential role in channel function, beyond inhibition of CDI and channel modulation.

**Figure 3:**
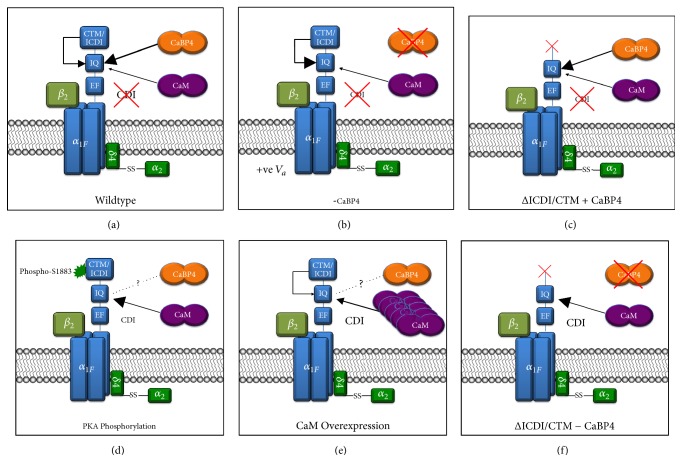
Mechanisms of C-terminal regulation. (a) In the wild-type Ca_V_1.4 channel, with CaM and CaBP4 coexpressed, CaBP4 and the ICDI/CTM competitively interact with the proximal C-terminus, preventing CaM-mediated calcium-dependent inactivation (CDI). (b) When CaBP4 is not present, the ICDI/CTM still competitively inhibits CaM-mediated CDI, but the interaction between the ICDI/CTM and proximal C-terminus causes a significant positive shift in the activation voltage. (c) In the absence of the ICDI/CTM, CaBP4 prevents CDI, and the half-maximal voltage is unaffected, suggesting an additional crucial role for the ICDI/CTM* in vivo*. (d) The ICDI/CTM can be phosphorylated at serine 1883 by protein kinase A (PKA), allowing CaM to facilitate CDI and modulate channel properties. (e) Overexpression of CaM results in CaM's outcompeting ICDI/CTM for binding to the proximal C-terminus, thus facilitating CDI. The effect of CaBP4 in this circumstance has yet to be investigated. (f) In the absence of both CaBP4 and the ICDI/CTM, CaM is not competitively inhibited from interaction with the proximal C-terminus; thus, it confers CDI.

Structural studies have shown that calcium-bound CaBP4 tightly binds the IQ-motif, thereby likely competitively inhibiting both CaM and ICDI/CTM binding, whereas calcium-free/Mg^2+^-bound CaBP4 does not. Therefore, Park et al. suggest that, with high intracellular calcium levels in darkness, CaBP4 simultaneously prevents CaM-mediated CDI and ICDI/CTM-mediated positive shifts in activation voltage, thus promoting the open state. In light, the decrease in cytosolic calcium destabilizes the CaBP4-IQ interaction, allowing for ICDI/CTM and/or CaM binding and rapid channel closure [76]. Alternatively, Yang et al. argue that the higher intracellular concentrations and binding affinity of CaM suggest an allosteric mechanism, and they provide compelling evidence that CaBP4 interactions with alternative, less competitive sites are sufficient for CaBP4 regulatory functions with channel-bound CaM [77]. Further research is required to determine how the complex interplays among CaM, CaBP4, the ICDI/CTM domain, and PKA phosphorylation of Ca_V_1.4 shapes the calcium current in photoreceptors for optimal visual function. For a summary of the effects of these interactions on CDI under various physiological circumstances, see Figure 3.

## 4. Ca_V_1.4 Dysfunction in Disease

### 4.1. Congenital Stationary Night Blindness 2 (CSNB2) Characteristics

Congenital stationary night blindness 2 (CSNB2, also known as incomplete CSNB) is an X-linked (CSNB2A) or autosomal (CSNB2B) recessive, minimally progressive disorder with substantial clinical heterogeneity. Symptoms may include nystagmus, strabismus, night blindness, photophobia, colour vision defects, and decreased visual acuity, the presence and severity of which vary greatly from patient to patient [78–80]. In fact, recent articles have emphasized that, given the low prevalence and impact of night vision problems in individuals with the condition (only affecting 54% in a recent analysis of 101 Dutch patients, [78, 81]), “night blindness” is a misnomer, suggesting “congenital rod-cone synaptic dysfunction” or the historical term “Åland Eye Disease” as viable alternatives [39, 82]. Electroretinography (ERG) and mutation analyses are the definitive tools for diagnosing CSNB2. Patients exhibit a characteristic reduction in scotopic b-wave amplitude, with preservation of the a-wave, indicating normal rod phototransduction with disrupted synaptic transmission [8, 38]. Photopic ERG (30 Hz-flicker) responses are also characteristically abnormal, making it possible to distinguish between CSNB2 and similar retinal afflictions including CSNB1, rod dystrophies, and cone dystrophies [83]. Observations in CSNB2 patients via optical coherence tomography (OCT) have revealed several anatomical abnormalities, including thinning of inner and outer retinal layers, foveal hypoplasia, reduced length of foveal cone outer segments, and suggested abnormal synaptogenesis [39, 84, 85]. Estimating the prevalence of CSNB2 is complicated by the aforementioned symptomatic variability, together with the historically rare use of electroretinography in common clinical practice. Nonetheless, a conservative estimate of 1 in 22,000 live-born males was calculated on the basis of the ratio of identified CSNB2-affected to all live-born males in Denmark between 1980 and 2009 [39]. Recent ERG analyses of heterozygous female carriers of* CACNA1F* mutations suggest that while the affected individuals lack subjective awareness, their objective visual function is mildly compromised [86, 87]. These data align with observations of heterozygous* Cacna1f*^+/−^ mice, which exhibit reduced visual function and mosaic abnormalities of retinal structure [86, 88]. It should also be noted that, despite the restricted expression of Ca_V_1.4, mutations in* CACNA1F* have been implicated in schizophrenia by haplotype-based haplotype relative (HHRR) analysis, but further work is needed to substantiate this correlation and determine whether the mutations cause or increase risk of the disorder [89].

### 4.2. CSNB2-Causing Mutations

Mutations associated with CSNB2 have been identified in two genes,* CACNA1F *and* CABP4*, which cause CSNB2A and CSNB2B, respectively [10, 11, 75]. Mutations in* CACNA1F* are more prevalent by far, being associated with >90% of CSNB2 cases [38]. Mutations in an additional gene,* CACNA2D4* (which codes for the *α*_2_*δ*_4_ subunit of Ca_V_1.4), have also been implicated in very rare cases of a CSNB2-like disorder, which may be reclassified as a CSNB2-subtype in time [21, 38, 90]. Given that CaBP4 and *α*_2_*δ*_4_ are important modulators of *α*_1F_/Ca_V_1.4 function, all CSNB2-causing mutations may cause disease by deleteriously affecting the photoreceptor presynaptic calcium current [17, 54]. In addition to CSNB2,* CACNA1F* mutations are also known to cause X-linked Cone-Rod Dystrophy 3 (CORDX3) [91, 92], Åland Eye Disease [39, 93, 94], and even a progressive retinal/optic disc atrophy [95]. Similarities between these allelic variants have led some to suggest that a degree of unification may be necessary [39].

While all known CSNB2-causing mutations affect presynaptic proteins associated with the photoreceptor calcium current, CSNB1 (also known as complete congenital stationary night blindness) is caused by deleterious mutations in essential postsynaptic proteins of ON-bipolar cells. Accordingly, these patients exhibit visual defects and ERG responses consistent with a loss of ON-bipolar cell function, but an intact OFF-bipolar cell pathway [38]. Though outside the scope of this review, studies of CSNB1 animal models have been valuable in determining the mechanisms underlying the ON-bipolar pathway (for a comprehensive review of CSNB1, and other CSNB subtypes, see Zeitz et al. [38]).

**Figure 4:**
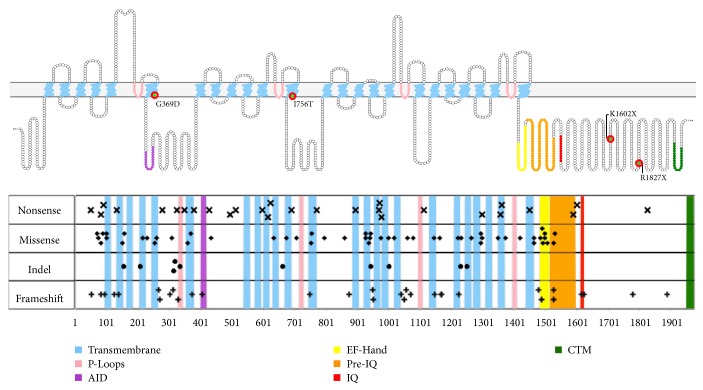
CSNB2A-causing mutations in* CACNA1F, *and their locations in the *α*_1F_ subunit (Isoform 1, NCBI Reference Sequences: NM_005183.3 (mRNA), NP_005174.2 (Protein), UNIPROT Identifier: O60840-1) (nomenclature of *α*_1F_ mutations differs between laboratories, as groups have used two distinct protein isoforms of different lengths (due to an 11 amino-acid difference in exon 9 beginning at residue 426). Consequently, mutations occurring after amino-acid 426 may be differentially annotated between publications (ex. R1827X [36] versus R1816X [37]). Readers should be aware of this discrepancy between publications, and authors should clearly indicate the particular isoform used when annotating mutations). Compiled from Zeitz et al. [38] and Hove et al. [39]. AID: alpha-interaction domain; CTM: C-terminal modulator/inhibitor of calcium-dependent inactivation. Mutations specifically addressed in the text are highlighted. *α*_1F_ topographical visualization generated with Protter [40].

Over 140 mutations in* CACNA1F *have been described (see Figure 4; for a detailed review of all known CSNB-causing mutations identified before 2015, see Zeitz et al. [38]). These have been insightfully divided into three categories: (1) loss-of-function mutations; (2) gain-of-function mutations; and (3) ICDI/C-terminal modulator- (CTM-) impairing mutations [36, 38, 39]. Diversity in the last two types of mutation certainly contributes to some of the clinical variability observed, but interestingly, substantial symptomatic variability is observed in patients with similar or even identical mutations [80, 96]. This may be attributable to the effects of other modulating genetic or environmental factors [79, 80, 96].

### 4.3. Effects of Mutations on Ca_V_1.4 Function

The effects of various CSNB2A-causing* CACNA1F* mutations on Ca_V_1.4 channel function have been investigated in several heterologous expression systems, in part to elucidate the pathogenic mechanisms contributing to CSNB2A variability. All nonsense mutations within 50–55 amino acids of a downstream splice site are expected to undergo nonsense-mediated decay, leading to a loss of Ca_V_1.4 channel expression, although several missense mutations have also been shown to abolish calcium influx with wild-type-like expression levels [36, 97–99]. Biochemical mechanisms underlying the loss of calcium influx with several of these point mutations are reviewed in Stockner and Koschak, 2013 [36]. Of the mutations characterized to date that cause significant changes in channel kinetics without abolishing measurable current, all cause a negative shift in activation voltage, which may cause a detrimental reduction in the dynamic range of photoreceptor responses or degeneration as a consequence of the sustained high levels of intracellular calcium. The most drastic of these, I745T, is associated with a particularly severe CSNB2A phenotype and significant retinal disruption [27, 28, 87, 100, 101]. The G369D mutation was reported by Hoda et al. to cause a significant (−13.7 mV) shift in activation voltage, in contrast to a previous report in which no such shift was found [23, 98]. The authors attributed this to electrostatic variability in vicinity of the mutation, due to slight differences in primary structure in the *α*_1F_ constructs that they used [23, 98]. This suggests that subtle electrostatic differences can drastically alter channel dynamics and may contribute to clinical variability in CSNB2A patients. The putative effect of R1827X, K1602X, and other C-terminal truncation mutations is the abolition of C-terminal regulation, discussed above* (C-Terminal Regulation and Calmodulin)*.

## 5. Insights from CSNB2A Animal Models

### 5.1. Overview of CSNB2 Models

Seven animal models with mutations in* Cacna1f* have been identified or created, revealing many insights into the physiological role of Ca_V_1.4. The first definitive model with a mutation in* Cacna1f* was published in 2005, with an insertion in exon 7 leading to a premature truncation at amino-acid G305. This* Cacna1f*^G305X^ mouse, in contrast to the human condition, exhibits a complete loss of both scotopic and photopic ERG b-waves [102]. To date, no behavioral or electrophysiological tests have revealed any visual function in the* Cacna1f*^G305X^ or in another loss-of-function mouse model,* Cacna1f*^Δ14–17^  (created by excision of exons 14–17, also leading to premature truncation) [103]. Visual function in these mice has been collectively evaluated by optokinetic response analysis [104], analysis of cortical visual evoked potentials [102], multiunit activity recording from the superior colliculus [102], electroretinography [100, 102], a visually guided water-maze behavioral task [86], and multielectrode array analyses of full-field flash ganglion cell responses in isolated retinas [105]. Similar results have been observed in two* “wait until dark” (wud) *zebrafish, which have premature truncations in* cacna1fa*, one of two zebrafish paralogs of human* CACNA1F*. These zebrafish have severely attenuated ERG a- and b-waves, exhibit no visual startle response, and do not follow visual stimuli, indicating that they are completely blind [106].

Why these models appear to lack detectable visual function while loss-of-function* CACNA1F* mutations in CSNB2-affected humans (~50% of known mutations) cause only a variable reduction in visual acuity (among other symptoms) is unclear. Expression of a second, compensatory voltage-gated calcium channel (most likely Ca_V_1.3) is the most plausible explanation, though this has yet to be shown definitively in the human retina. In* Cacna1d*-KO (*α*_1D_/Ca_V_1.3) mice, there is no observed change in performance in visually guided behavioral tasks, but a mild decrease in photoreceptor ribbon synapse density and disrupted ribbon morphology has been reported by two groups. ERG analyses of retinas in these mice, however, have provided conflicting reports. A recent study of* Cacna1d*-KO mice reported decreased ERG a-wave, b-wave, and oscillatory potential (associated with amacrine cell function) amplitudes, in contrast to a previous study in which no such reductions were found [30, 107]. ERG analyses of* Cacna1f*/*Cacna1d* double-KO mice showed no significant differences between these and* Cacna1f*-KO mice [108]. These discrepancies warrant further investigation into the role of Ca_V_1.3 in the photoreceptor synaptic terminal.

In contrast to mouse and zebrafish models, premature truncation of* Cacna1f* in a spontaneous rat model* (Cacna1f*^R981X^) causes impaired but not absent visual function, as well as a reported effect on several nonvisual behaviors [109, 110]. Interestingly, this model also exhibits decreased skeletal muscle endurance and contractility, leading the authors to suggest a role for Ca_V_1.4 outside of the retina and immunocytes in rat [111].

Two additional mouse models of CSNB2 with unique mutations also have been reported and shown to exhibit unique phenotypic characteristics. The* nob2 (no b-wave 2)* mouse was originally discovered as part of an ERG-based screen at the Jackson Laboratory for spontaneous retinal mutants and predicted to cause a complete loss of Ca_V_1.4 expression due to the insertion of a transposable element in* Cacna1f* exon 2 [112, 113]. Differences in phenotype between this mouse and the previously described* Cacna1f*^G305X^ knockout model, including an observable ERG b-wave in* nob2 *[112, 114], led to the identification of an alternate* Cacna1f* splice variant, which results in reduced (~10%) expression of wild-type-like Ca_V_1.4 in the* nob2* retina [115]. The photopic and scotopic contrast sensitivities of the optokinetic response in* nob2* mice are only slightly below those of wild-type, suggesting that low-level expression of *α*_1F_ is sufficient for near-optimal visual function, despite the persistence of other morphological anomalies in these retinas [104, 115, 116].

The* Cacna1f*^I756T^ mouse represents the only* Cacna1f* missense mutant model described to date. This mutant was engineered to emulate a CSNB2-causing human mutation, discovered in a New Zealand family with particularly severe clinical characteristics, including nonocular neurological abnormalities and visual defects in heterozygous female carriers (rarely reported with* CACNA1F* mutations)^101^. Electrophysiological analysis of the resultant Ca_V_1.4 channel in a heterologous system revealed a drastic change in the activation voltage, leading to a reduced dynamic range (described above:* Effects of Mutations on Ca*_V_*1.4 Function*). ERG recordings from* Cacna1f*^I756T^ mice and CSNB2 patients carrying the analogous mutation are similarly affected (unlike other* Cacna1f *mutant models), with a residual but significantly attenuated b-wave, reduced a-wave, and other characteristics generally absent from other CSNB2A models [28, 101]. Interestingly, Knoflach et al. have reported an upregulation of* Cacna1d *mRNA (*α*_1D_/Ca_V_1.3) in the* Cacna1f*^I756T^ mouse retina, providing some animal evidence for the mechanism that might contribute to residual vision in human CSNB2A patients [28].

### 5.2. Physiological and Morphological Observations in CSNB2A Model Retinas

Animal models have also allowed for extensive descriptions of physiological changes in response to mutations in* Cacna1f*. In retinas of all* Cacna1f*-KO models, synaptic ribbons of the photoreceptor synaptic terminals (discussed above:* Ca*_V_*1.4 Function: The Ribbon Synapse and Phototransduction*) never anchor to the presynaptic membrane or elongate into their characteristic morphology, according to electron microscopical and immunohistochemical observations [27, 106, 117, 118]. Comparisons to* CaBP4*-KO and* Cacna1f*^I756T^ retinas suggest that the synthesis of *α*_1F_ protein and the assembly of complete Ca_V_1.4 channels in the membrane (regardless of kinetics) are necessary for the initial formation of photoreceptor presynaptic terminals, until P13 after which biophysical properties become important for proper maturation as these models exhibit typical retinal development until eye opening. Ca_V_1.4 may play a scaffolding role for synaptic elements, including PSD-95 and PMCA, which help develop the presynaptic ribbon [27].

Recent insights from mouse models support this scaffolding role for Ca_V_1.4, particularly in rod synaptogenesis. Ca_V_1.4 appears to coordinate the membrane localization of the extracellular protein ELFN1, which associates specifically with the *α*_2_*δ*_4_ subunit of the channel. ELFN1 is a trans-synaptic protein, which facilitates synaptic alignment with the metabotropic glutamate receptor mGluR6 in rod ON-bipolar cells. In the absence of Ca_V_1.4, the *α*_2_*δ*_4_ subunit, or ELFN1 itself, rod synapses do not form. Remarkably, neither *α*_2_*δ*_4_ nor ELFN1 is necessary for cone synaptogenesis; this suggests that cones may use an alternative, as yet undiscovered, molecular mechanism for synaptogenesis [58, 119].

The disruption of the presynaptic terminal and the absence or irregularity of calcium signaling causes complete failure (in loss-of-function [KO]) or severe aberration (in I756T) of synaptogenesis between the photoreceptors and second-order neurons (bipolar and horizontal cells) [27, 102, 117, 118]. In response, normally rod-connected dendrites of both bipolar and horizontal cells form abnormal extensions into the outer nuclear layer (“sprouting”), and photoreceptor terminals have been reported to retract from the outer plexiform layer into the outer nuclear layer [100, 116, 118]. Despite their abnormal course and destination, sprouted dendrites have been reported to form ectopic synapses in the ONL as revealed by their close apposition to photoreceptor processes containing any of several presynaptic elements, including Bassoon [100, 102, 118], RIBEYE [27, 88, 118], Cplx4 [86, 118], vGlut1 [86, 118], Cplx3 [86], Piccolo [88, 100], VAMP2 [118], and synaptophysin [118]. Despite the presence of vesicle-release machinery at these ectopic synapse-like associations, no evidence of synaptic transmission has been reported to date [118]. Abnormal bipolar cell synapses in the ONL of a human CSNB2A patient have also been suggested from abnormalities observed using optical coherence tomography [84]. Comparisons to animal models of other retinal diseases suggest that bipolar and horizontal cell dendrites may sprout to seek contact with active presynaptic cells. In* CNGA3*-KO mice, in which cone phototransduction is disrupted, cone bipolar cells form ectopic synapses with nearby rods. In* rhodopsin-*KO mice, in which rod phototransduction is disrupted, rod bipolar cells form ectopic synapses with cones. In* CNGA3*^−/−^*/Rho*^−/−^ double-KO mice, no ectopic synapses are observed by P46 [120].

**Figure 5:**
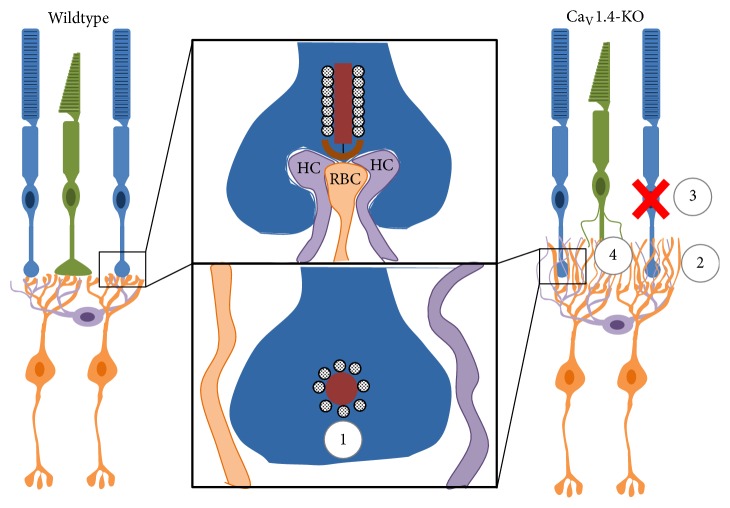
Known morphophysiological abnormalities in* Cacna1f*-KO mouse retinas (lacking Ca_V_1.4). (1) The synaptic ribbon fails to mature or anchor to the presynaptic membrane; (2) normally rod-contacting bipolar and horizontal cell dendrites sprout past the outer plexiform layer, into the outer nuclear layer, potentially seeking presynaptic partners; (3) photoreceptors (both rods and cones) gradually die, leading to loss of nuclei in the outer nuclear layer; and (4) cone photoreceptors exhibit aberrant axonal morphology, including axonal sprouting.

CSNB2A is commonly referred to as a nonprogressive (“stationary”) condition. However, both* Cacna1f*-KO and* Cacna1f*^I756T^  mice exhibit progressive photoreceptor dystrophy and degeneration. Histological images showing thinning of the ONL and TUNEL-labeling of presumably apoptotic cells, as well as photoreceptor-specific immunolabeling suggest that photoreceptors are gradually lost in all CSNB2A mouse models, with* Cacna1f*^I756T^  retinas exhibiting the most rapid degeneration [86, 100, 117, 118]. These data are supported by longitudinal ERG analyses, which have shown significant declines in a-wave amplitudes between one and eight months in both mutants, but much more dramatic reduction in* Cacna1f*^I756T^  retinas [100]. Immunohistochemical visualization of adult cones reveals general dystrophy, absent (KO) or abnormal (I756T) terminals, and axonal abnormalities including varicosities and sprouting [86, 88, 100, 117, 118]. We have recently shown that sprouted PKC*α*+ rod bipolar cells form ectopic contacts with these dystrophic cones in the* Cacna1f*-KO retina, which increasingly consolidate as these mice age [88]. These contacts, along with the cone axonal sprouting (which have not been described in any other synaptopathy model), are particularly intriguing, as they suggest that many cones retain the potential for synaptic plasticity beyond the normal period of photoreceptor synaptogenesis [88, 118]. For a summary of known major morphophysiological abnormalities in the mouse* Cacna1f*-KO retina, see Figure 5.

The phenotype of* Cacna1f*-KO mice makes this model uniquely suited to answer an outstanding question in retinal neuroscience: can photoreceptors form functional synapses with inner retinal neurons, after the normal period of development has ceased? This question is especially pertinent now, following the recent evidence that transplanted photoreceptor precursors do not integrate into the circuitry of degenerating retinas, as was originally believed [121]. Photoreceptors in* Cacna1f*-KO retinas are apparently “invisible” to second-order neurons, yet the inner retina exhibits few of the degenerative changes observed in models of primarily photoreceptor degeneration [122]. Therefore,* Cacna1f*-KO animal models may be useful tools for identifying the factors that are critical for normal photoreceptor synaptogenesis, and further investigation of these models may offer, not only insight into potential clinical interventions for patients with CSNB2A, but also hope for the millions living with photoreceptor-destroying degenerative retinal diseases.

## 6. Conclusion

Ca_V_1.4 is a highly specialized voltage-gated calcium channel that has evolved unique characteristics for its distinct role within the nervous system. The remarkable sensitivity of the visual system is dependent on Ca_V_1.4, as evidenced by the vision losses in patients with CSNB2A and other clinical conditions caused by mutations in* CACNA1F *(e.g., Åland Eye Disease); accumulating evidence suggests that this channel may also serve yet unknown or poorly understood functional roles in other systems. Research into the biophysical effects of mutations, in* CACNA1F *and mechanisms of Ca_V_1.4 regulation and gating, has been enlightening for our understanding of voltage-gated calcium channels, CSNB2A, and retinal circuitry as a whole. In particular, the recent use of animal models to investigate physiological changes in response to known CSNB2A-causing mutations has provided a wealth of knowledge into the functional roles of Ca_V_1.4 (and other VGCCs) in the nervous system. Continuing to unearth the complexities of neural circuitry, by exploiting these models as unique and powerful research tools, brings us closer to devising life-changing clinical therapies for CSNB2A and other retinal degenerative diseases.
